# Supplementary material for: DMRforPairs: identifying Differentially Methylated Regions between unique samples using array based methylation profiles
Source: BMC Bioinformatics. 2014 May 15;15:141. doi: 10.1186/1471-2105-15-141 (PMC4046028; doi:10.1186/1471-2105-15-141)
Supplement: Additional file 2 — DMRforPairs output for the comparison of A431-MCF7 and NA17018-NA17105. Please start from the HTML files in each folder. Available via the BMC Bioinformatics website. [file 1471-2105-15-141-S2.zip › 1394847754114233_MOESM2_ESM/A431_MCF7/figures/14437.pdf]

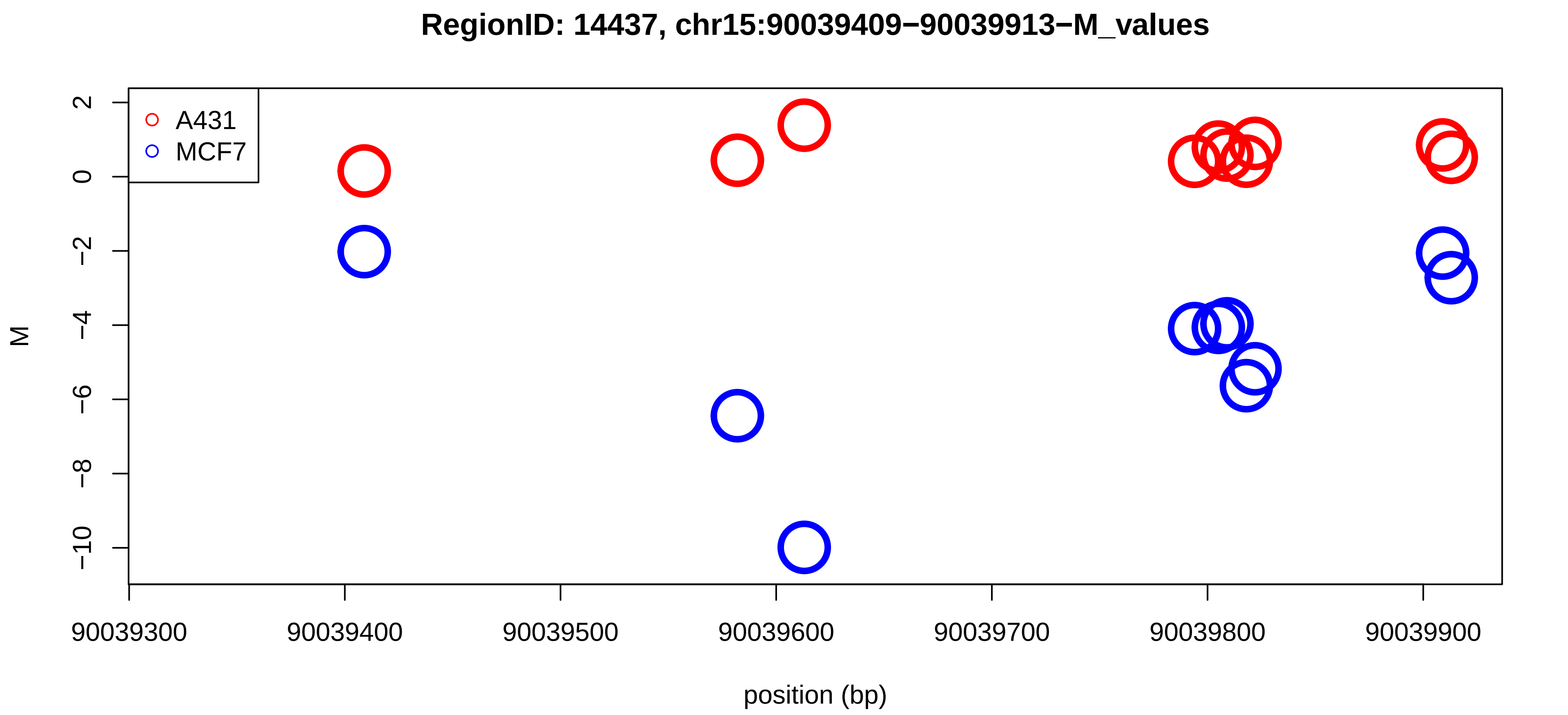

RegionID: 14437, chr15:90039409−90039913−M_values
A431
MCF7
M
2
0
−2
−4
−6
−8
−10
90039300
90039400
90039500
90039600
90039700
90039800
90039900
position (bp)


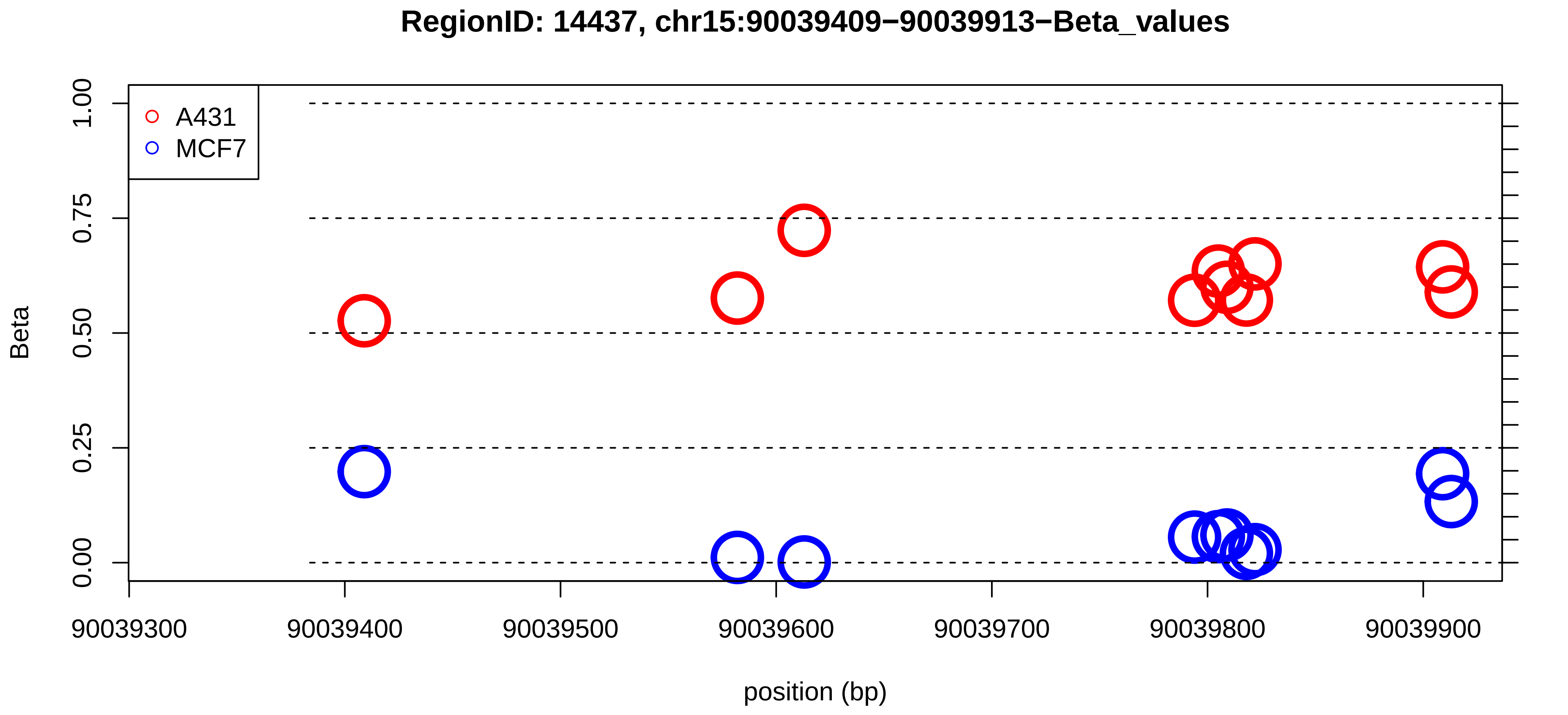

RegionID: 14437, chr15:90039409−90039913−Beta_values
A431
MCF7
Beta
1.00
0.75
0.50
0.25
0.00
90039300
90039400
90039500
90039600
90039700
90039800
90039900
position (bp)

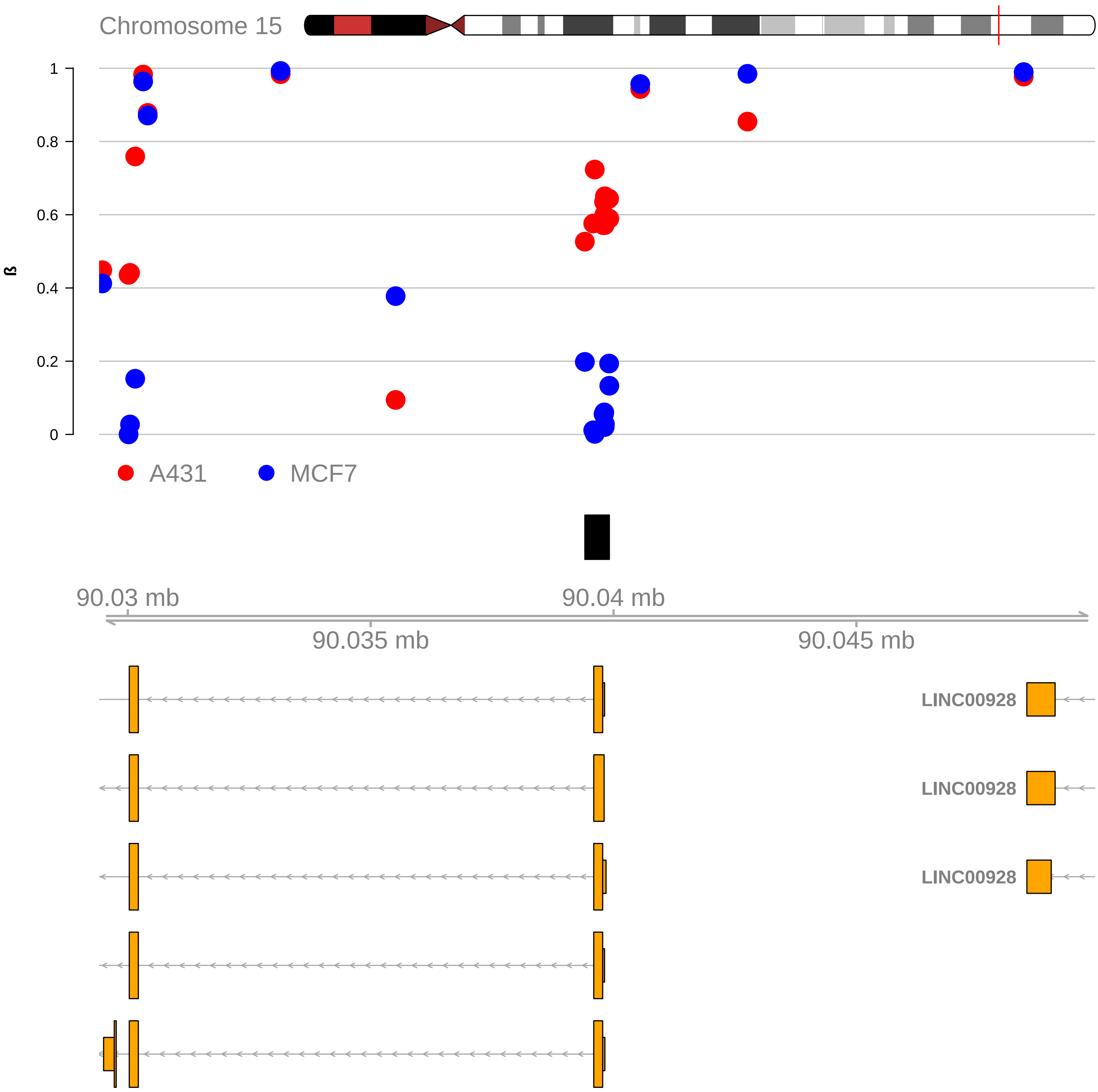

Chromosome 15
1
0.8
0.6
0.4
0.2
0
ß
A431
MCF7
90.03 mb
90.04 mb
90.035 mb
90.045 mb
LINC00928
LINC00928
LINC00928
